# Modeling Temperature-Dependent Vibration Damping in C/SiC Fiber-Reinforced Ceramic-Matrix Composites

**DOI:** 10.3390/ma13071633

**Published:** 2020-04-01

**Authors:** Longbiao Li

**Affiliations:** College of Civil Aviation, Nanjing University of Aeronautics and Astronautics, No.29 Yudao St., Nanjing 210016, China; llb451@nuaa.edu.cn; Tel.: +86-25-8489-5963

**Keywords:** ceramic-matrix composites (CMCs), C/SiC, damping, temperature-dependent, matrix cracking, interface debonding

## Abstract

In this paper, the temperature-dependent vibration damping in C/SiC fiber-reinforced ceramic-matrix composites (CMCs) with different fiber preforms under different vibration frequencies is investigated. A micromechanical temperature-dependent vibration damping model is developed to establish the relationship between composite damping, material properties, internal damage mechanisms, and temperature. The effects of fiber volume, matrix crack spacing, and interface properties on temperature-dependent composite vibration damping of CMCs and interface damage are analyzed. The experimental temperature-dependent composite damping of 2D and 3D C/SiC composites is predicted for different loading frequencies. The damping of the C/SiC composite increases with temperature to the peak value and then decreases with temperature. When the vibration frequency increases from f = 1 to 10 Hz, the peak value of composite damping and corresponding temperature both decrease due to the decrease of interface debonding and slip range, and the damping of 2D C/SiC is much higher than that of 3D C/SiC at temperature range from room temperature to 400 °C. When the fiber volume and interface debonding energy increase, the peak value of composite damping and the corresponding temperature decreases, mainly attributed to the decrease of interface debonding and slip range.

## 1. Introduction

Ceramic-matrix composites (CMCs) are the candidate materials for hot section components of aerospace vehicles, high thrust-to-weight-ratio aeroengines, satellite attitude control engines, ramjets, and thermal protection systems [1,2]. However, in the above applications, there exist vibration and noise problems. Failure analysis of rockets and satellites shows that about two-thirds of the failures are related to vibration and noise, leading to reduced operational control accuracy, structural fatigue damage, and shortened safety life [3]. Therefore, studying the damping performance of CMCs and improving their reliability in the service environment of vibration and noise is an important guarantee for the safe service of CMCs in various fields [4].

Compared with metals and alloys, CMCs have many unique damping mechanisms due to their internal structure and complex damage mechanisms [5,6,7,8]. The damping properties of composites are usually much more complicated than homogenous material. Temperature, moisture, loading frequency, and wave form affect the damping of composites [9,10]. During manufacturing and service, cracks might occur in the matrix, fiber, and interface both between fiber/matrix and neighboring plies [11]. Friction slip in the interface debonding region among matrix crack space consumes energy [12]. The internal friction of CMCs is affected by fabrication method [10], interphase thickness [13], oxidation [14], coating, and heat treatment [15,16]. Holmes and Cho [17] developed an analytical model for predicting energy dissipation of SiC/CAS-II during a cycle based on the interfacial friction slip mechanisms. The energy dissipation corresponding to each cycle depends on stress level, matrix crack spacing, and interface frictional shear stress. Li [18] investigated internal frictional behavior of C/SiC considering fiber failure and developed temperature- and time-dependent damage models for matrix cracking [19,20]. The dynamic properties extracted from vibration response of damaged composites can be used for damage monitoring, and these include natural frequencies, mode shape, and damping. Li [21] established a relationship between natural frequency, critical rotation speed, and internal damage inside CMCs. Kyriazoglou et al. [22] measured and analyzed the specific damping capacity (SDC) of composite beams in flexure before and after quasi-static loading or fatigue damage. Zhang and Hartwig [23] detected a damping plateau from fatigue cycles in epoxy composites due to energy balance between fatigue load input and damage dissipation. However, in the research mentioned above, the synergistic effects of vibration frequency and fiber preform on temperature-dependent vibration damping of fiber-reinforced CMCs was not established.

In this paper, a micromechanical vibration damping model is developed to analyze the temperature-dependent damping of C/SiC composites with different fiber preforms under different loading frequencies. The relationships between composite damping, internal damage, and temperature are established considering different material properties and damage states. The experimental temperature-dependent damping of 2D and 3D C/SiC under vibration frequencies of f = 1, 2, 5, and 10 Hz is predicted.

## 2. Temperature-Dependent Damping Models

When a solid vibrates, its kinetic and strain energies transform mutually. The largest strain energy, equaling the entire energy driving vibration, determines the intensities of deformation or vibration of the structure. The proportion of energy consumed during one vibration cycle is directly associated with the vibration attenuation rate, which is also known as damping. The composite damping is given by: [7]

(1) $$\eta =\frac{U_{d}}{2\pi U}$$

where *U*_d_ and *U* are dissipated energy density and maximum strain energy per cycle, respectively.

For CMCs without damage, the temperature-dependent composite damping (*η*_a_) is obtained as:

(2) $$\eta_{a}\left(T\right)=\frac{E_{f}\left(T\right)V_{f}\eta_{f}+E_{m}\left(T\right)V_{m}\eta_{m}}{E_{f}\left(T\right)V_{f}+E_{m}\left(T\right)V_{m}}$$

where *η*_f_ and *η*_m_ denote fiber and matrix damping, respectively; *V*_f_ and *V*_m_ are the volume of fiber and matrix, respectively; and *E*_f_(*T*) and *E*_m_(*T*) are the temperature-dependent elastic modulus of fiber and matrix, respectively.

When damage occurs inside of CMCs, the effective temperature-dependent matrix elastic modulus (${\bar{E}}_{m}\left(T\right)$) is obtained as:

(3) $${\bar{E}}_{m}\left(T\right)=\frac{\tau_{i}\left(T\right)}{V_{m}}\frac{1}{\frac{\tau_{i}\left(T\right)}{E_{c}\left(T\right)}+\frac{r_{f}}{4l_{c}\left(T\right)}\frac{\Delta \sigma }{E_{f}\left(T\right)}{\left(\frac{V_{m}E_{m}\left(T\right)}{V_{f}E_{c}\left(T\right)}\right)}^{2}}-\frac{V_{f}E_{f}\left(T\right)}{V_{m}}$$

where τ_i_(*T*) is the temperature-dependent interface shear stress; *r*_f_ is fiber radius; *E*_c_(*T*) is the temperature-dependent longitudinal modulus of intact composite material; *l*_c_(*T*) is the temperature-dependent matrix crack spacing; and Δ*σ* is applied stress range (Δ*σ* = 2*σ*).

(4) $$\sigma_{c}=\sigma \left(1+\sin\omega t\right)$$

where *ω* is a vibration frequency.

For damaged CMCs, the energy dissipation during each vibration cycle contributes to the composite damping (*η*_b_), which is given by:

(5) $$\eta_{b}\left(T\right)=\frac{U_{d}\left(T\right)}{2\pi U\left(T\right)}$$

where

(6) $$U_{d}\left(T\right)=U_{d\_u}\left(T\right)+U_{d\_r}\left(T\right)$$

(7) $$U\left(T\right)=U_{f}\left(T\right)+U_{m}\left(T\right)$$

where *U*_d_u_(*T*) and *U*_d_r_(*T*) are the temperature-dependent dissipated energy density upon unloading and reloading, respectively, and *U*_f_(*T*) and *U*_m_(*T*) are the temperature-dependent fiber and matrix strain energy density, respectively.

(8) $$U_{d\_u}\left(T\right)=2\pi r_{f}\tau_{i}\left(T\right)\left[\frac{\Delta \sigma }{V_{f}E_{f}\left(T\right)}l_{y}^{2}\left(T\right)-\frac{8}{3}\frac{E_{c}\left(T\right)}{V_{m}E_{f}\left(T\right)E_{m}\left(T\right)}\frac{\tau_{i}\left(T\right)}{r_{f}}l_{y}^{3}\left(T\right)\right]$$

(9) $$U_{d\_r}\left(T\right)=2\pi r_{f}\tau_{i}\left(T\right)\left[\frac{\Delta \sigma }{V_{f}E_{f}\left(T\right)}l_{z}^{2}\left(T\right)-\frac{8}{3}\frac{E_{c}\left(T\right)}{V_{m}E_{f}\left(T\right)E_{m}\left(T\right)}\frac{\tau_{i}\left(T\right)}{r_{f}}l_{z}^{3}\left(T\right)\right]$$

(10) $$\begin{array}{ll} U_{f}\left(T\right)= & \pi r_{f}^{2}\{\frac{\sigma^{2}}{V_{f}^{2}E_{f}\left(T\right)}l_{d}\left(T\right)-2\frac{\sigma \tau_{i}\left(T\right)}{r_{f}V_{f}E_{f}\left(T\right)}l_{d}^{2}\left(T\right)+\frac{4}{3}\frac{\tau_{i}^{2}\left(T\right)}{r_{f}^{2}E_{f}\left(T\right)}l_{d}^{3}\left(T\right)+\frac{\sigma_{\mathrm{fo}}^{2}\left(T\right)}{E_{f}\left(T\right)}\left(\frac{l_{c}\left(T\right)}{2}-l_{d}\left(T\right)\right)\\ & +\frac{2r_{f}\sigma_{\mathrm{fo}}\left(T\right)}{\rho E_{f}\left(T\right)}\left(\frac{V_{m}}{V_{f}}\sigma_{\mathrm{mo}}\left(T\right)-2\frac{l_{d}\left(T\right)}{r_{f}}\tau_{i}\left(T\right)\right)\left(1-\exp\left(-\rho \frac{l_{c}\left(T\right)/2-l_{d}\left(T\right)}{r_{f}}\right)\right)\\ & +\frac{r_{f}}{2\rho E_{f}\left(T\right)}{\left(\frac{V_{m}}{V_{f}}\sigma_{\mathrm{mo}}\left(T\right)-2\frac{l_{d}\left(T\right)}{r_{f}}\tau_{i}\left(T\right)\right)}^{2}\left(1-\exp\left(-2\rho \frac{l_{c}\left(T\right)/2-l_{d}\left(T\right)}{r_{f}}\right)\right)\} \end{array}$$

(11) $$\begin{array}{ll} U_{m}\left(T\right)= & \pi r_{f}^{2}\{\frac{4}{3}\frac{V_{f}^{2}\tau_{i}^{2}\left(T\right)}{r_{f}^{2}V_{m}^{2}E_{m}\left(T\right)}l_{d}^{3}\left(T\right)+\frac{\sigma_{\mathrm{mo}}^{2}}{E_{m}}\left(\frac{l_{c}\left(T\right)}{2}-l_{d}\left(T\right)\right)\\ & -\frac{2r_{f}\sigma_{\mathrm{mo}}\left(T\right)}{\rho E_{m}\left(T\right)}\left(\sigma_{\mathrm{mo}}\left(T\right)-2\tau_{i}\left(T\right)\frac{V_{f}}{V_{m}}\frac{l_{d}\left(T\right)}{r_{f}}\right)\left[1-\exp\left[-\frac{\rho \left(l_{c}\left(T\right)/2-l_{d}\left(T\right)\right)}{r_{f}}\right]\right]\\ & +\frac{r_{f}}{2\rho E_{m}\left(T\right)}{\left(\sigma_{\mathrm{mo}}\left(T\right)-2\tau_{i}\left(T\right)\frac{V_{f}}{V_{m}}\frac{l_{d}\left(T\right)}{r_{f}}\right)}^{2}\left[1-\exp\left[-2\frac{\rho \left(l_{c}\left(T\right)/2-l_{d}\left(T\right)\right)}{r_{f}}\right]\right]\} \end{array}$$

where *l*_d_(*T*), *l*_y_(*T*), and *l*_z_(*T*) are temperature-dependent interface debonding length, counter slip length, and new slip length, respectively; *ρ* is shear-lag model parameter; and *σ*_fo_(*T*) and *σ*_mo_(*T*) are temperature-dependent fiber and matrix axial stress in the interface bonding region, respectively.

(12) $$l_{d}\left(T\right)=\frac{r_{f}}{2}\left(\frac{V_{m}E_{m}\left(T\right)\sigma }{V_{f}E_{c}\left(T\right)\tau_{i}\left(T\right)}-\frac{1}{\rho }\right)-\sqrt{{\left(\frac{r_{f}}{2\rho }\right)}^{2}+\frac{r_{f}V_{m}E_{m}\left(T\right)E_{f}\left(T\right)}{E_{c}\left(T\right)\tau_{i}^{2}\left(T\right)}\xi_{d}\left(T\right)}$$

(13) $$l_{y}\left(T\right)=\frac{1}{2}\left\{l_{d}\left(T\right)-\left[\frac{r_{f}}{2}\left(\frac{V_{m}E_{m}\left(T\right)\sigma }{V_{f}E_{c}\left(T\right)\tau_{i}\left(T\right)}-\frac{1}{\rho }\right)-\sqrt{{\left(\frac{r_{f}}{2\rho }\right)}^{2}+\frac{r_{f}V_{m}E_{m}\left(T\right)E_{f}\left(T\right)}{E_{c}\left(T\right)\tau_{i}{}^{2}\left(T\right)}\xi_{d}\left(T\right)}\right]\right\}$$

(14) $$l_{z}\left(T\right)=l_{y}\left(T\right)-\frac{1}{2}\left\{l_{d}\left(T\right)-\left[\frac{r_{f}}{2}\left(\frac{V_{m}E_{m}\left(T\right)\sigma }{V_{f}E_{c}\left(T\right)\tau_{i}\left(T\right)}-\frac{1}{\rho }\right)-\sqrt{{\left(\frac{r_{f}}{2\rho }\right)}^{2}+\frac{r_{f}V_{m}E_{m}\left(T\right)E_{f}\left(T\right)}{E_{c}\left(T\right)\tau_{i}{}^{2}\left(T\right)}\xi_{d}\left(T\right)}\right]\right\}$$

(15) $$\sigma_{\mathrm{fo}}\left(T\right)=\frac{E_{f}\left(T\right)}{E_{c}\left(T\right)}\sigma +E_{f}\left(T\right)\left(\alpha_{\mathrm{lc}}\left(T\right)-\alpha_{\mathrm{lf}}\left(T\right)\right)\Delta Τ$$

(16) $$\sigma_{\mathrm{mo}}\left(T\right)=\frac{E_{m}\left(T\right)}{E_{c}\left(T\right)}\sigma +E_{m}\left(T\right)\left(\alpha_{\mathrm{lc}}\left(T\right)-\alpha_{\mathrm{lm}}\left(T\right)\right)\Delta Τ$$

where *ξ*_d_(*T*) denotes temperature-dependent interface debonding energy; *α*_lf_(*T*), *α*_lm_(*T*), and *α*_lc_(*T*) are temperature-dependent fiber, matrix, and composite axial thermal expansion coefficient, respectively; and ΔT denotes temperature difference between testing temperature (*T*) and fabricated temperature (*T*_0_).

The total temperature-dependent composite damping (*η*_c_) can be determined as:

(17) $$\eta_{c}=\eta_{a}+\eta_{b}$$

where *η*_a_ and *η*_b_ can be determined by Equations (2) and (5), respectively.

## 3. Results and Discussion

The material properties of the C/SiC composite are given by: *V*_f_ = 0.3, *r*_f_ = 3.5 μm, *ξ*_d_ = 0.1 J/m^2^, *η*_f_ = 0.002, *η*_m_ = 0.001, and *T*_0_ = 1000 °C, and the temperature-dependent constituent properties are given by [24,25,26,27,28]:

(18) $$E_{f}\left(T\right)=230\left[1-2.86\times 10^{-4}\exp\left(\frac{T+273}{324}\right)\right],T<2000\;{}^{\circ}C$$

(19) $$E_{m}\left(T\right)=\frac{350}{460}\left[460-0.04\left(T+273\right)\exp\left(-\frac{962}{T+273}\right)\right],T\in \left[27^{{}^{\circ}}C,\;1500\;{}^{\circ}C\right]$$

(20) $$\alpha_{\mathrm{lf}}\left(T\right)=2.529\times 10^{-2}-1.569\times 10^{-4}\left(T+273\right)+2.228\times 10^{-7}{\left(T+273\right)}^{2}\;-1.877\times 10^{-11}{\left(T+273\right)}^{3}-1.288\times 10^{-14}{\left(T+273\right)}^{4},T\in \left[27^{{}^{\circ}}C,\;2227\;{}^{\circ}C\right]$$

(21) $$\alpha_{\mathrm{rf}}\left(T\right)=-1.86\times 10^{-1}+5.85\times 10^{-4}\left(T+273\right)-1.36\times 10^{-8}{\left(T+273\right)}^{2}\;+1.06\times 10^{-22}{\left(T+273\right)}^{3},T\in \left[27^{{}^{\circ}}C,\;2500\;{}^{\circ}C\right]$$

(22) $$\alpha_{\mathrm{lm}}\left(T\right)=\alpha_{\mathrm{rm}}\left(T\right)=\left\{\begin{array}{c} -1.8276+0.0178\left(T+273\right)-1.5544\times 10^{-5}{\left(T+273\right)}^{2}\\ \;\;\;\;\;\;\;\;\;\;\;\;\;\;\;+4.5246\times 10^{-9}{\left(T+273\right)}^{3},\;\;T\in \left[0^{{}^{\circ}}C,\;1000\;{}^{\circ}C\right]\\ 5.0\times 10^{-6},\;\;T>1000\;{}^{\circ}C \end{array}\right.$$

(23) $$\tau_{i}\left(T\right)=\tau_{0}+\mu \frac{\left|\alpha_{\mathrm{rf}}\left(T\right)-\alpha_{\mathrm{rm}}\left(T\right)\right|\left(T_{0}-T\right)}{A}$$

where τ_0_ is the steady-state interface shear stress; *μ* is the interface frictional coefficient; *α*_rf_ and *α*_rm_ denote the temperature-dependent fiber and matrix radial thermal expansion coefficient, respectively; and *A* is a constant depending on the elastic properties of the matrix and the fiber.

(24) $$\xi_{d}\left(T\right)=\xi_{\mathrm{dr}}\left[1-\frac{\int_{T_{r}}^{T}C_{P}\left(T\right)dT}{\int_{T_{r}}^{T_{0}}C_{P}\left(T\right)dT}\right]$$

where *T*_r_ denotes the reference temperature; *T*_0_ denotes the fabricated temperature; and *ξ*_dr_ denotes the interface debonding energy at the reference temperature of *T*_r_.

The effects of material properties and damage state on temperature-dependent composite damping and interface damage of the C/SiC composite are analyzed.

### 3.1. Effect of Fiber Volume on Temperature-Dependent Damping of C/SiC Composite

**Figure 1:**
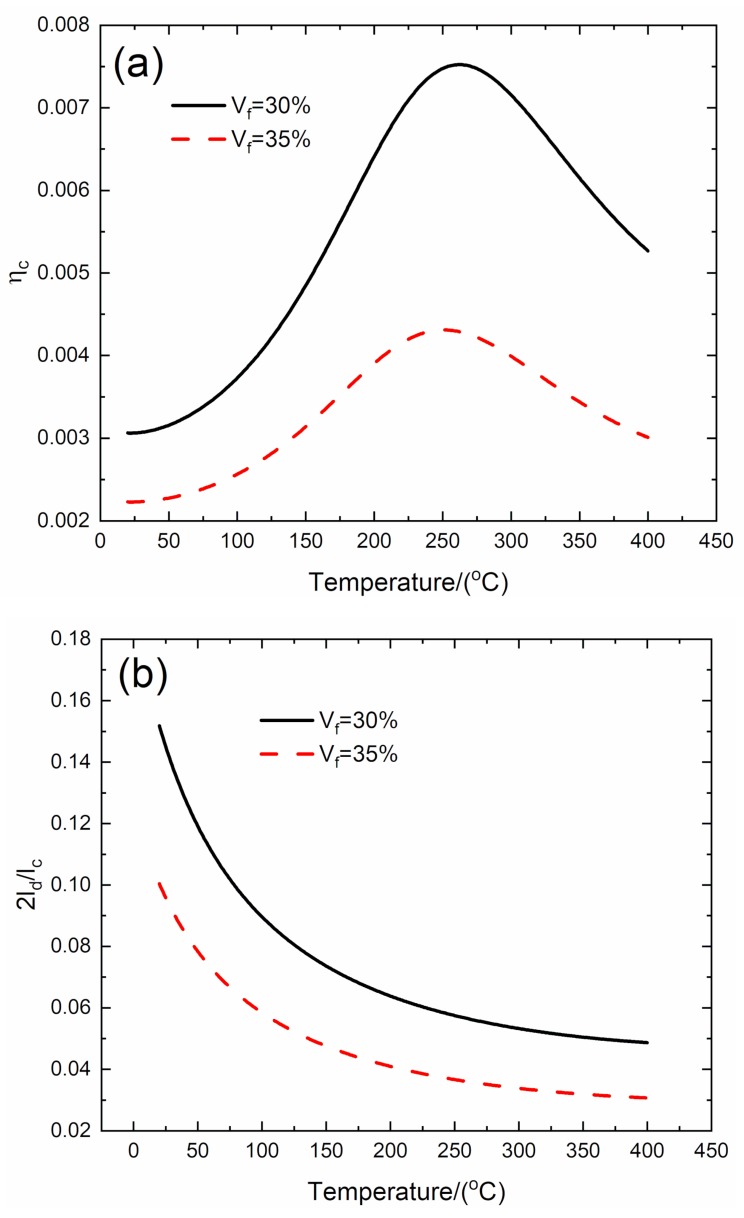

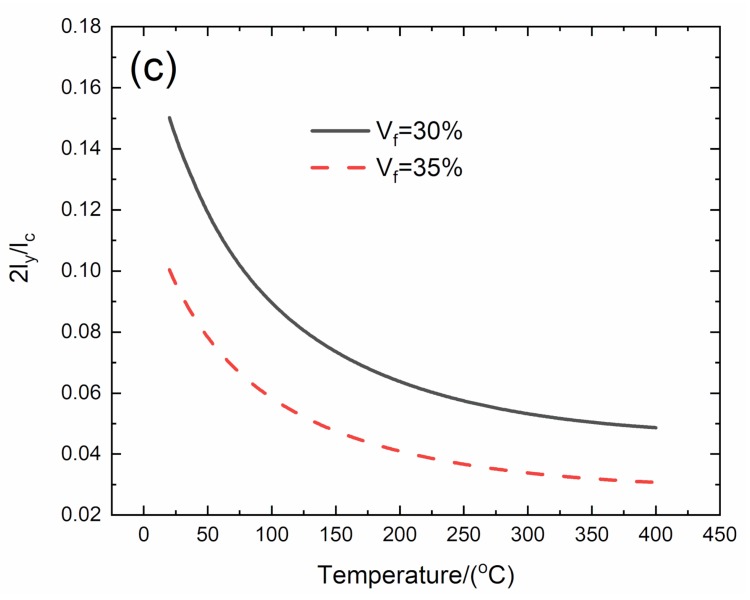
Effect of fiber volume (*V*_f_ = 30% and 35%) on (**a**) the temperature-dependent composite damping (*η*_c_) versus temperature curves; (**b**) the temperature-dependent interface debonding length (2*l*_d_/*l*_c_) versus temperature curves; and (**c**) the temperature-dependent interface slip length (2*l*_y_/*l*_c_) versus temperature curves of C/SiC composite.

The effect of fiber volume (*V*_f_ = 30% and 35%) on temperature-dependent composite damping (*η*_c_), interface debonding, and slip length (2*l*_d_/*l*_c_, 2*l_y_*/*l*_c_) versus temperature curves of the C/SiC composite is analyzed for the temperature range from room temperature (*T* = 20 °C) to elevated temperature of *T* = 400 °C, as shown in Figure 1 and Table 1.

When *V*_f_ = 30%, the temperature-dependent composite damping (*η*_c_) increases from *η*_c_ = 0.00306 at *T* = 20 °C to peak value *η*_c_ = 0.00752 at *T* = 262 °C and decreases to *η*_c_ = 0.00527 at *T* = 400 °C; the temperature-dependent interface debonding length (2*l*_d_/*l*_c_) decreases from 2*l*_d_/*l*_c_ = 0.152 at *T* = 20 °C to 2*l*_d_/*l*_c_ = 0.048 at *T* = 400 °C; and the temperature-dependent interface slip length (2*l*_y_/*l*_c_) decreases from 2*l*_y_/*l*_c_ = 0.15 at *T* = 20 °C to 2*l*_y_/*l*_c_ = 0.048 at *T* = 400 °C.

When *V*_f_ = 35 %, the temperature-dependent composite damping (*η*_c_) increases from *η*_c_ = 0.00223 at *T* = 20 °C to peak value *η*_c_ = 0.00431 at *T* = 250 °C, and decreases to *η*_c_ = 0.00301 at *T* = 400 °C; the temperature-dependent interface debonding length (2*l*_d_/*l*_c_) decreases from 2*l*_d_/*l*_c_ = 0.1 at *T* = 20 °C to 2*l*_d_/*l*_c_ = 0.03 at *T* = 400 °C; and the temperature-dependent interface slip length (2*l*_y_/*l*_c_) decreases from 2*l*_y_/*l*_c_ = 0.1 at *T* = 20 °C to 2*l*_y_/*l*_c_ = 0.03 at *T* = 400 °C.

At the temperature range from room temperature (*T* = 20 °C) to elevated temperature of *T* = 400 °C, the temperature-dependent composite damping of the C/SiC composite increases with temperature to the peak value first and then decreases with temperature. When the fiber volume increases from *V*_f_ = 30% to 35%, the temperature-dependent peak value damping of the C/SiC composite (*η*_c_) decreases from *η*_c_ = 0.00752 to *η*_c_ = 0.00431, and the corresponding temperature for the peak value damping of the C/SiC composite decreases from *T* = 262 °C to *T* = 250 °C, mainly attributed to the decrease of interface debonding and slip length.

### 3.2. Effect of Matrix Crack Spacing on Temperature-Dependent Damping of C/SiC Composite

**Figure 2:**
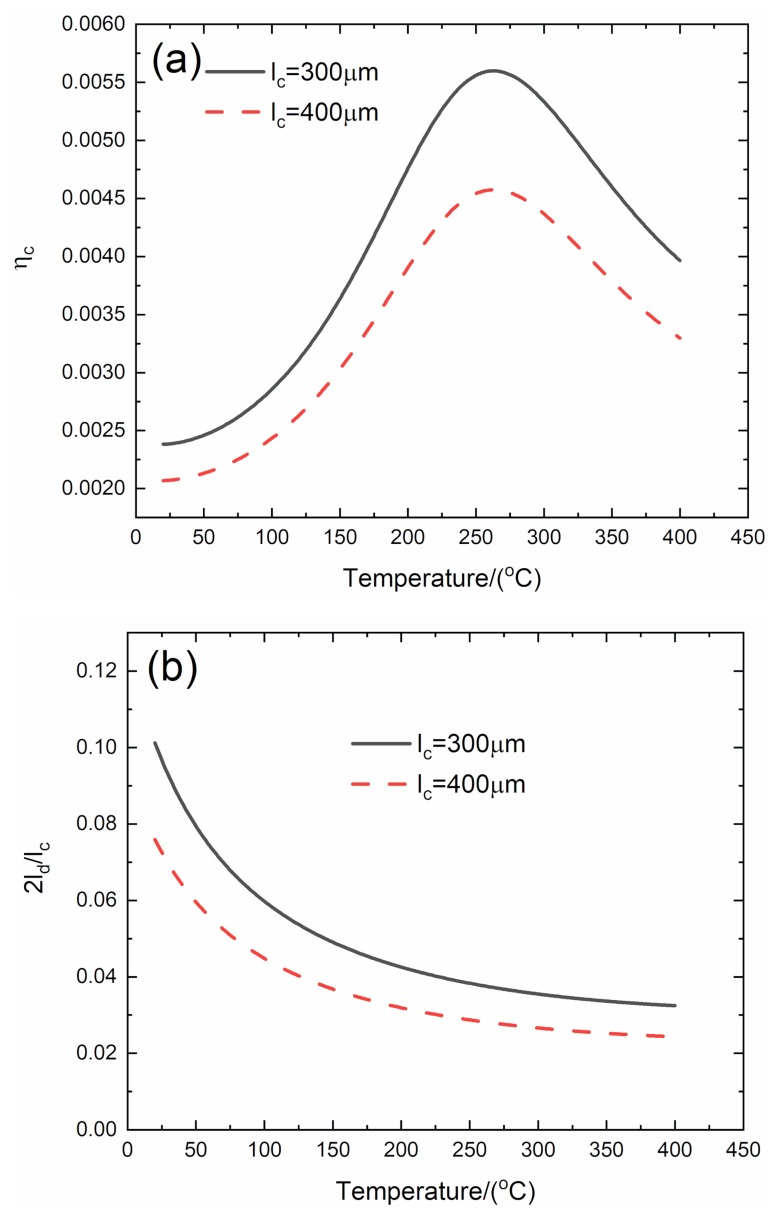

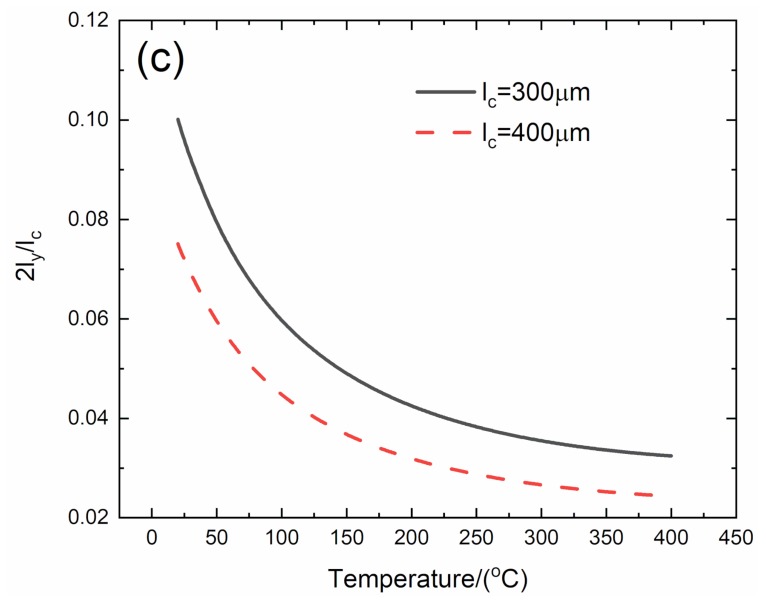
Effect of matrix crack spacing (*l*_c_ = 300 and 400 μm) on (**a**) the temperature-dependent composite damping (*η*_c_) versus temperature curves; (**b**) the temperature-dependent interface debonding length (2*l*_d_/*l*_c_) versus temperature curves; and (**c**) the temperature-dependent interface slip length (2*l*_y_/*l*_c_) versus temperature curves of C/SiC composite.

The effect of matrix crack spacing (*l*_c_ = 300 and 400 μm) on temperature-dependent composite damping (*η*_c_), interface debonding, and slip length (2*l*_d_/*l*_c_, 2*l*_y_/*l*_c_) versus temperature curves of the C/SiC composite is analyzed for the temperature range from room temperature (*T* = 20 °C) to elevated temperature of *T* = 400 °C, as shown in Figure 2 and Table 2.

When *l*_c_ = 300 μm, the temperature-dependent composite damping (*η*_c_) increases from *η*_c_ = 0.00238 at *T* = 20 °C to peak value *η*_c_ = 0.0056 at *T* = 263 °C and decreases to *η*_c_ = 0.00397 at *T* = 400 °C; the temperature-dependent interface debonding length (2*l*_d_/*l*_c_) decreases from 2*l*_d_/*l*_c_ = 0.101 at *T* = 20 °C to 2*l*_d_/*l*_c_ = 0.032 at *T* = 400 °C; and the temperature-dependent interface slip length (2*l*_y_/*l*_c_) decreases from 2*l*_y_/*l*_c_ = 0.1 at *T* = 20 °C to 2*l*_y_/*l*_c_ = 0.032 at *T* = 400 °C.

When *l*_c_ = 400 μm, the temperature-dependent composite damping (*η*_c_) increases from *η*_c_ = 0.00207 at *T* = 20 °C to peak value *η*_c_ = 0.00458 at *T* = 263 °C and decreases to *η*_c_ = 0.00207 at *T* = 400 °C; the temperature-dependent interface debonding length (2*l*_d_/*l*_c_) decreases from 2*l*_d_/*l*_c_ = 0.0759 at *T* = 20 °C to 2*l*_d_/*l*_c_ = 0.0243 at *T* = 400 °C; and the temperature-dependent interface slip length (2*l*_y_/*l*_c_) decreases from 2*l*_y_/*l*_c_ = 0.0751 at *T* = 20 °C to 2*l*_y_/*l*_c_ =0.0243 at *T* = 400 °C.

When matrix crack spacing increases from *l*_c_ = 300 to 400 μm, the peak damping of the C/SiC composite decreases from *η*_c_ = 0.0056 to *η*_c_ = 0.00458, and the interface debonding and slip length at the same temperature also decrease.

### 3.3. Effect of Interface Debonding Energy on Temperature-Dependent Damping of C/SiC Composite

**Figure 3:**
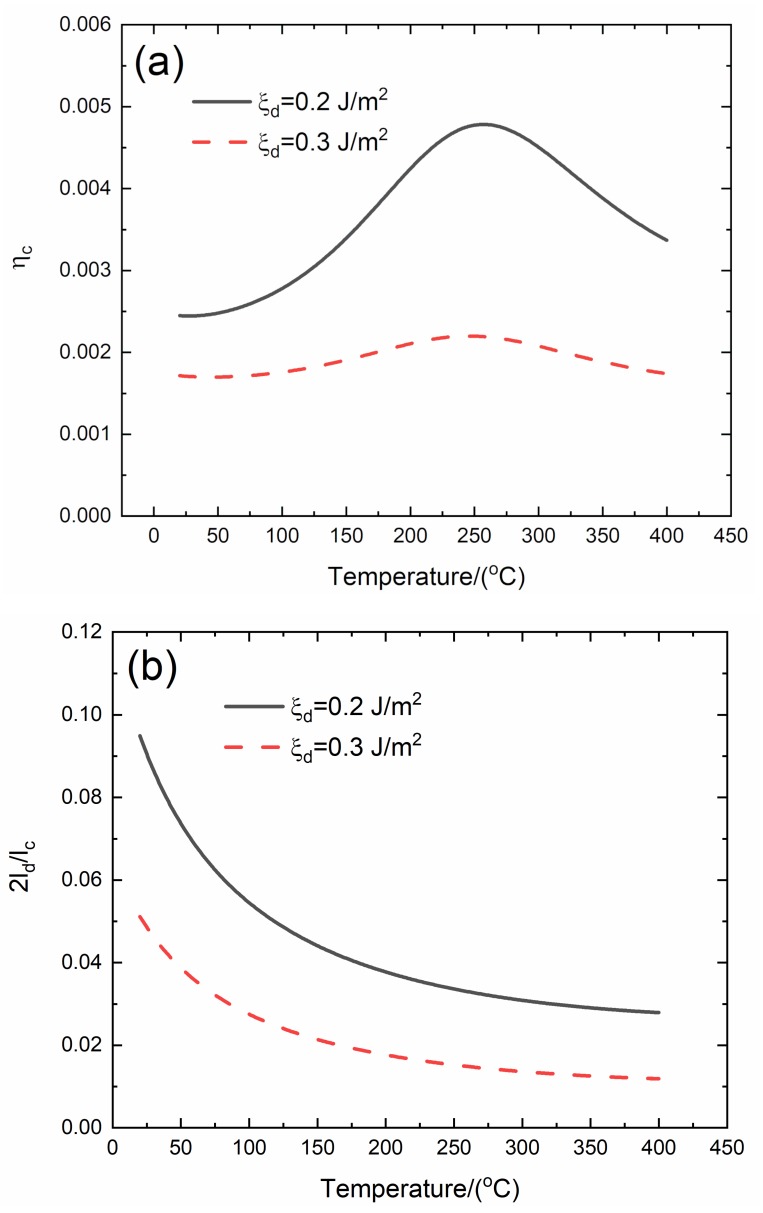

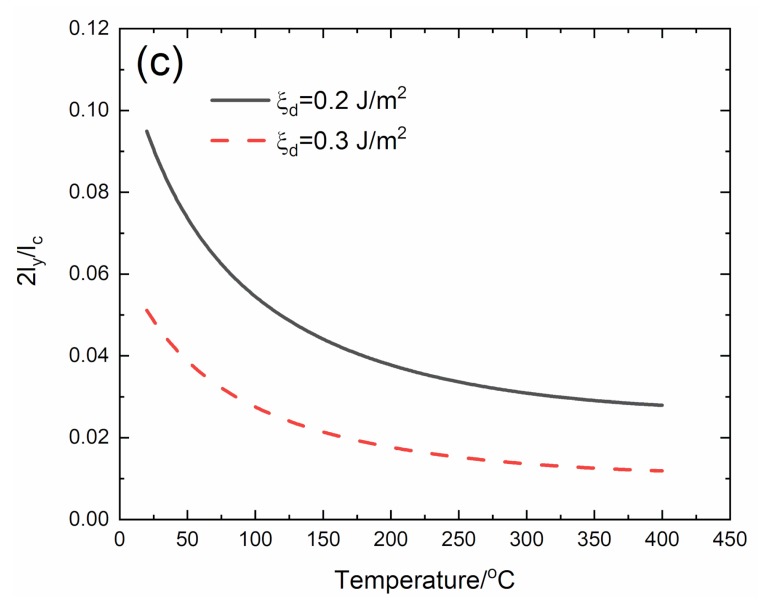
Effect of interface debonding energy (*ξ*_d_ = 0.2 and 0.3 J/m^2^) on (**a**) the temperature-dependent composite damping (*η*_c_) versus temperature curves; (**b**) the temperature-dependent interface debonding length (2*l*_d_/*l*_c_) versus temperature curves; and (**c**) the temperature-dependent interface slip length (2*l*_y_/*l*_c_) versus temperature curves of C/SiC composite.

The effect of interface debonding energy (*ξ*_d_ = 0.2 and 0.3 J/m^2^) on temperature-dependent composite damping (*η*_c_), interface debonding, and slip length (2*l*_d_/*l*_c_, 2*l*_y_/*l*_c_) versus temperature curves of the C/SiC composite is analyzed for the temperature range from room temperature (*T* = 20 °C) to elevated temperature of *T* = 400 °C, as shown in Figure 3 and Table 3.

When *ξ*_d_ = 0.2 J/m^2^, the temperature-dependent composite damping (*η*_c_) increases from *η*_c_ = 0.00245 at *T* = 20 °C to peak value *η*_c_ = 0.00478 at *T* = 256 °C and decreases to *η*_c_ = 0.00337 at *T* = 400 °C; the temperature-dependent interface debonding length (2*l*_d_/*l*_c_) decreases from 2*l*_d_/*l*_c_ = 0.0949 at *T* = 20 °C to 2*l*_d_/*l*_c_ = 0.0279 at *T* = 400 °C; and the temperature-dependent interface slip length (2*l*_y_/*l*_c_) decreases from 2*l*_y_/*l*_c_ = 0.0949 at *T* = 20 °C to 2*l*_y_/*l*_c_ = 0.0279 at *T* = 400 °C.

When *ξ*_d_ = 0.3 J/m^2^, the temperature-dependent composite damping (*η*_c_) increases from *η*_c_ = 0.00172 at *T* = 20 °C to peak value *η*_c_ = 0.0022 at *T* = 245 °C and then decreases to *η*_c_ = 0.00174 at *T* = 400 °C; the temperature-dependent interface debonding length (2*l*_d_/*l*_c_) decreases from 2*l*_d_/*l*_c_ = 0.0511 at *T* = 20 °C to 2*l*_d_/*l*_c_ = 0.0118 at *T* = 400 °C; and the temperature-dependent interface slip length (2*l*_y_/*l*_c_) decreases from 2*l_y_*/*l*_c_ = 0.0511 at *T* = 20 °C to 2*l*_y_/*l*_c_ = 0.0118 at *T* = 400 °C.

When interface debonding energy increases from *ξ*_d_ =0.2 to 0.3 J/m^2^, the peak damping of the C/SiC composite decreases from *η*_c_ =0.00478 to *η*_c_ =0.0022, and the corresponding temperature for peak damping decreases from *T* = 256 to *T* =245 °C, and the interface debonding and slip length at the same temperature also decrease.

### 3.4. Effect of Steady-State Interface Shear Stress on Temperature-Dependent Damping of C/SiC Composite

**Figure 4:**
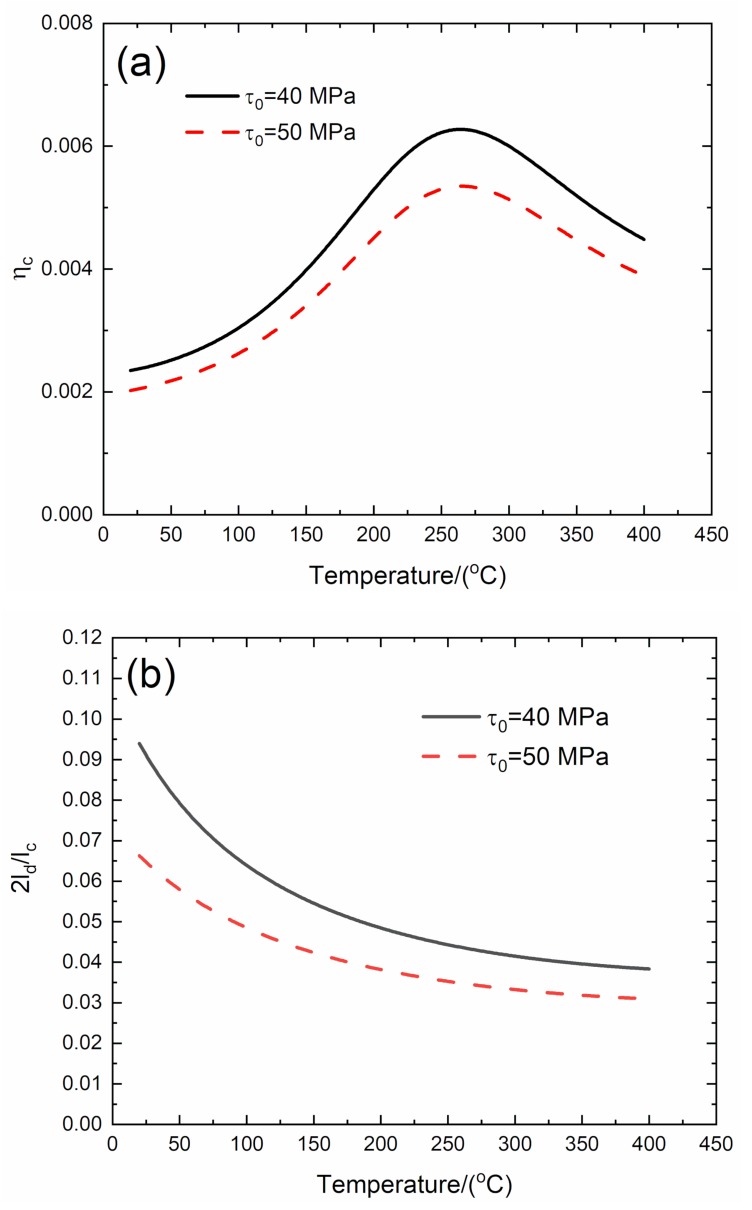

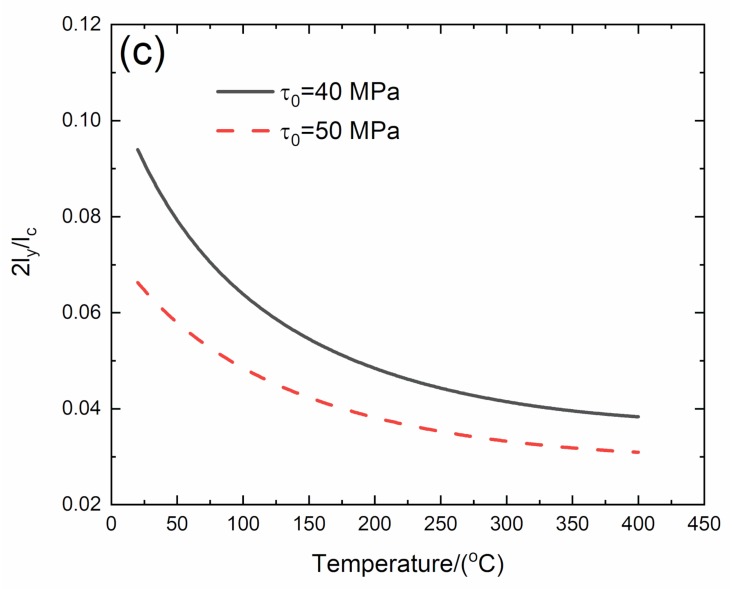
Effect of steady-state interface shear stress (*τ*_0_ = 40 and 50 MPa) on (**a**) the temperature-dependent composite damping (*η*_c_) versus temperature curves; (**b**) the temperature-dependent interface debonding length (2*l*_d_/*l*_c_) versus temperature curves; and (**c**) the temperature-dependent interface slip length (2*l*_y_/*l*_c_) versus temperature curves of C/SiC composite.

The effect of steady-state interface shear stress (*τ*_0_ = 40 and 50 MPa) on temperature-dependent composite damping (*η*_c_), interface debonding, and slip length (2*l*_d_/*l*_c_, 2*l*_y_/*l*_c_), and interface slip length versus temperature curves of the C/SiC composite are analyzed for the temperature range from room temperature (*T* = 20 °C) to elevated temperature of *T* = 400 °C, as shown in Figure 4 and Table 4.

When *τ*_0_ = 40 MPa, the temperature-dependent composite damping (*η*_c_) increases from *η*_c_ = 0.00235 at *T* = 20 °C to peak value *η*_c_ = 0.00627 at *T* = 264 °C and decreases to *η*_c_ = 0.00448 at *T* = 400 °C; the interface debonding length (2*l*_d_/*l*_c_) decreases from 2*l*_d_/*l*_c_ = 0.094 at *T* = 20 °C to 2*l*_d_/*l*_c_ = 0.038 at *T* = 400 °C; and the interface slip length (2*l*_y_/*l*_c_) decreases from 2*l*_y_/*l*_c_ = 0.094 at *T* = 20 °C to 2*l*_y_/*l*_c_ = 0.038 at *T* = 400 °C.

When *τ*_0_ = 50 MPa, the temperature-dependent composite damping (*η*_c_) increases from *η*_c_ = 0.00202 at *T* = 20 °C to peak value *η*_c_ = 0.00535 at *T* = 265 °C and decreases to *η*_c_ = 0.00389 at *T* = 400 °C; the temperature-dependent interface debonding length (2*l*_d_/*l*_c_) decreases from 2*l*_d_/*l*_c_ = 0.0663 at *T* = 20 °C to 2*l*_d_/*l*_c_ = 0.0309 at *T* = 400 °C; and the temperature-dependent interface slip length (2*l*_y_/*l*_c_) decreases from 2*l_y_*/*l*_c_ = 0.0663 at *T* = 20 °C to 2*l*_y_/*l*_c_ = 0.0309 at *T* = 400 °C.

When the steady-state interface shear stress increases from *τ*_0_ = 40 to 50 MPa, the peak damping of the C/SiC composite decreases from *η*_c_ = 0.00627 to *η*_c_ = 0.00535, and the interface debonding and slip length at the same temperature also decrease.

## 4. Experimental Comparisons

Wang et al. [15] investigated damping capacity of 2D and 3D T-300^TM^ C/SiC composites at different vibration frequencies. The 2D C/SiC composite is prepared by laminating 1K T-300 woven carbon fabrics, and the 3D C/SiC composite is prepared by braiding 3K T-300 carbon fibers in a four-step method. The volume of fiber was about 40% and the fiber diameter is 7.0 μm. The C/SiC with the PyC interphase was fabricated using chemical vapor infiltration (CVI). The deposition conditions of PyC interlayer were as follows: temperature 960 °C, pressure 5 kPa, Ar flow 200 ml/min, and butane flow 15 ml/min. The infiltration conditions of the SiC matrix were as follows: temperature 1000 °C, pressure 5 kPa, time 120 h, H_2_ flow 350 ml/min, Ar flow 350 ml/min, and molar ratio of H_2_ and MTS 10. A Dynamical Mechanical Analyzer (DMA 2980) made by TA company, USA, was used for damping measurements of the C/SiC composite. All of the measurements were performed in air atmosphere from room temperature to 400 °C, and the testing frequencies were f = 1, 2, 5, and 10 Hz.

### 4.1. 2D C/SiC Composite

#### 4.1.1. *f* = 1 Hz

**Figure 5:**
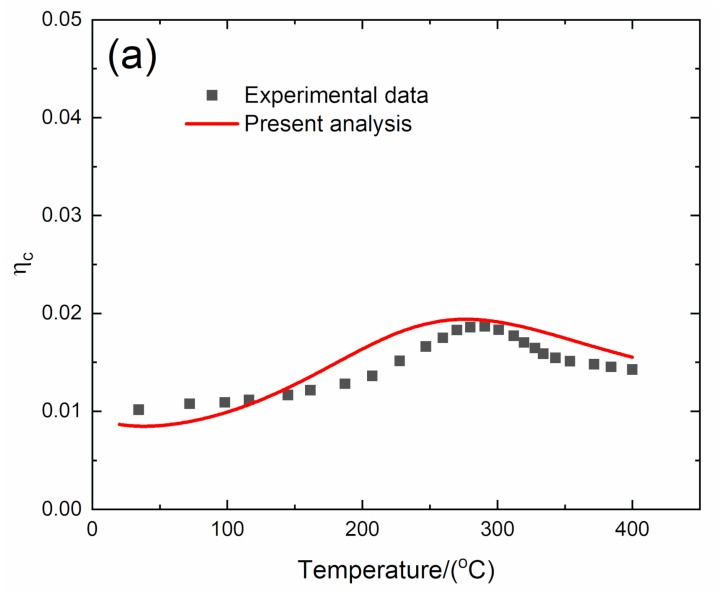

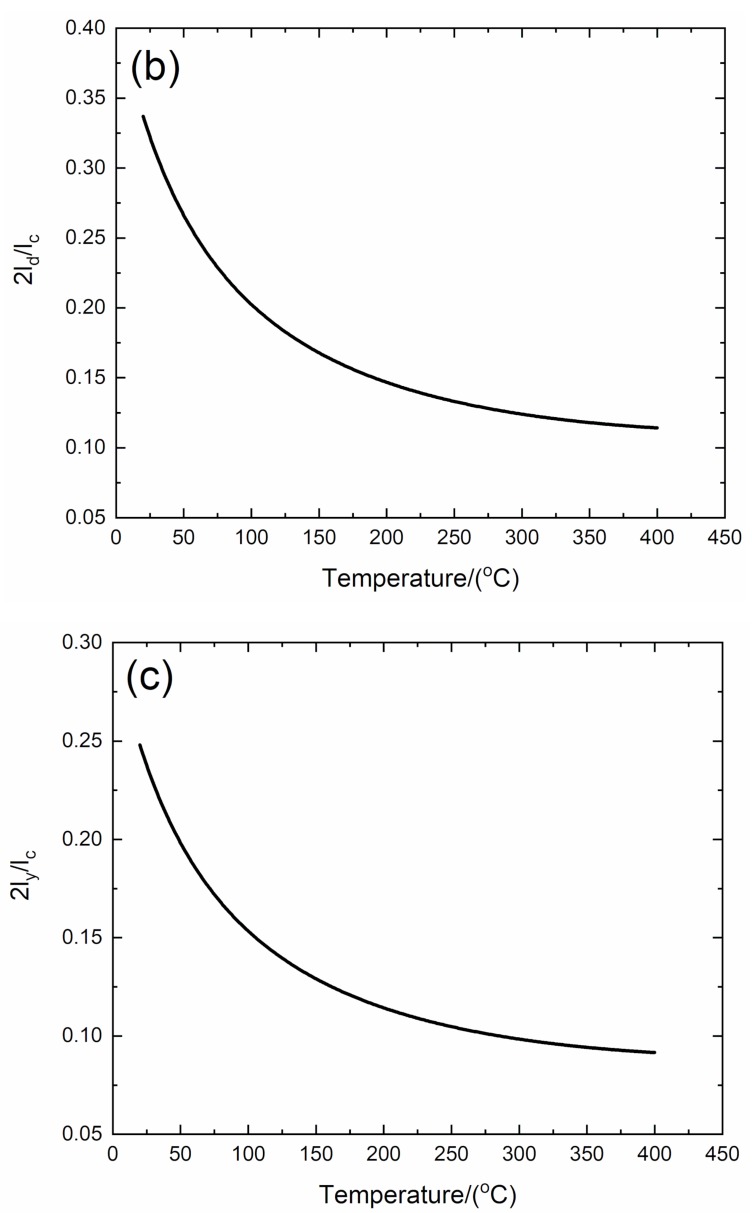
(**a**) Experimental and predicted temperature-dependent composite damping (*η*_c_) versus temperature curves; (**b**) the temperature-dependent interface debonding length (2*l*_d_/*l*_c_) versus temperature curves; and (**c**) the temperature-dependent interface slip length (2*l*_y_/*l*_c_) versus temperature curves of 2D C/SiC composite at the vibration frequency of *f* = 1 Hz.

The experimental and predicted temperature-dependent composite damping (*η*_c_), interface debonding, and slip length (2*l*_d_/*l*_c_, 2*l*_y_/*l*_c_) versus temperature curves of the 2D C/SiC composite at the vibration frequency of *f* = 1 Hz are shown in Figure 5 and Table 5. The predicted peak composite damping agrees with experimental data, and the predicted corresponding temperature for peak composite damping is a little lower than the experimental data.

The experimental composite damping increases from *η*_c_ = 0.01 at room temperature to peak value of *η*_c_ = 0.019 at temperature of *T* = 283 °C and then decreases to *η*_c_ = 0.014 at temperature of *T* = 400 °C. The theoretical predicted composite damping increases from *η*_c_ = 0.008 at room temperature to peak value *η*_c_ = 0.019 at temperature of *T* = 279 °C and then decreases to *η*_c_ = 0.015 at temperature of *T* = 400 °C. The interface debonding length (2*l*_d_/*l*_c_) decreases from 2*l*_d_/*l*_c_ = 0.337 at room temperature to 2*l*_d_/*l*_c_ = 0.114 at temperature of *T* = 400 °C, and the interface slip length (2*l*_y_/*l*_c_) decreases from 2*l*_y_/*l*_c_ = 0.248 at room temperature to 2*l*_y_/*l*_c_ = 0.091 at temperature of *T* = 400 °C.

#### 4.1.2. *f* = 2 Hz

**Figure 6:**
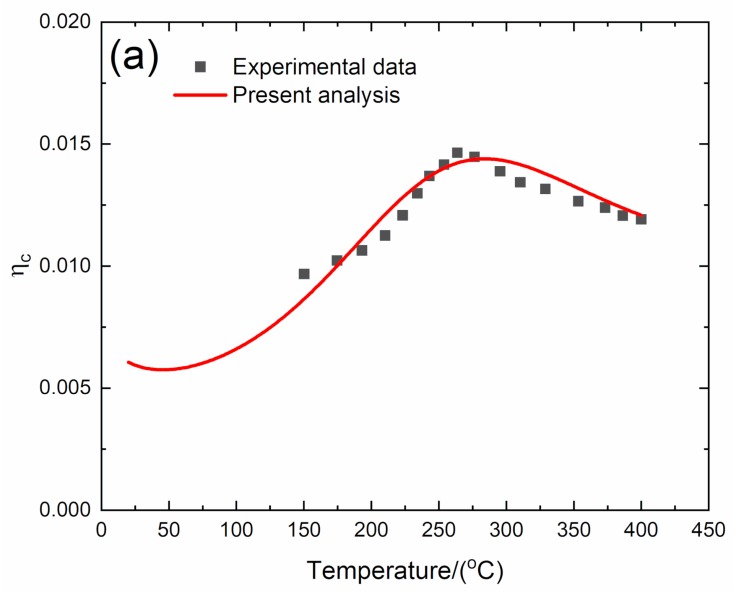

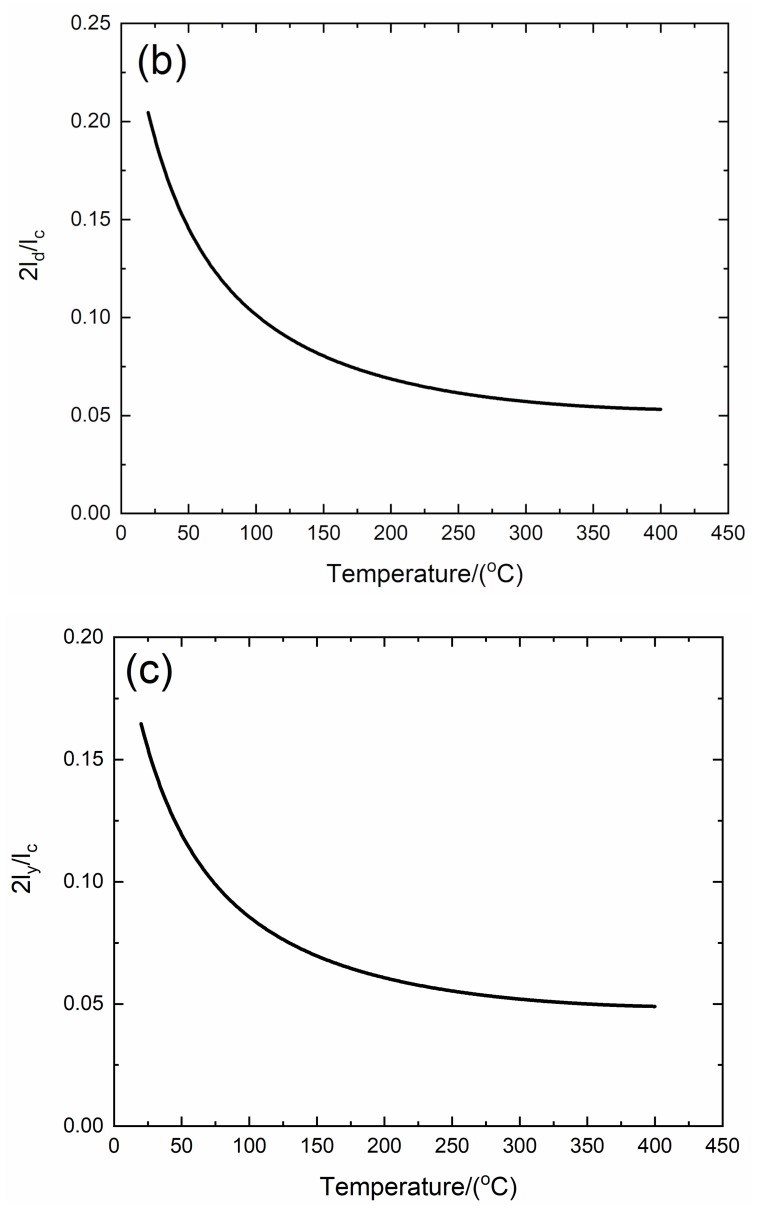
(**a**) Experimental and predicted temperature-dependent composite damping (*η*_c_) versus temperature curves; (**b**) the temperature-dependent interface debonding length (2*l*_d_/*l*_c_) versus temperature curves; and (**c**) the temperature-dependent interface slip length (2*l*_y_/*l*_c_) versus temperature curves of 2D C/SiC composite at the vibration frequency of *f* = 2 Hz.

The experimental and predicted temperature-dependent composite damping (*η*_c_), interface debonding, and slip length (2*l*_d_/*l*_c_, 2*l*_y_/*l*_c_) versus temperature curves of the 2D C/SiC composite at the vibration frequency of *f* = 2 Hz are shown in Figure 6 and Table 5.

The experimental composite damping (*η*_c_) increases from *η*_c_ = 0.009 at temperature of *T* = 150 °C to the peak value of *η*_c_ = 0.015 at temperature of *T* = 266 °C and then decreases to *η*_c_ = 0.012 at temperature of *T* = 400 °C. The theoretical predicted composite damping increases from *η*_c_ = 0.006 at room temperature to the peak value of *η*_c_ = 0.0144 at temperature of *T* = 283 °C and then decreases to *η*_c_ = 0.012 at temperature of *T* = 400 °C. The interface debonding length (2*l*_d_/*l*_c_) decreases from 2*l*_d_/*l*_c_ = 0.2 at room temperature to 2*l*_d_/*l*_c_ = 0.05 at temperature of *T* = 400 °C, and the interface slip length (2*l*_y_/*l*_c_) decreases from 2*l*_y_/*l*_c_ = 0.16 at room temperature to 2*l*_y_/*l*_c_ = 0.048 at temperature of *T* = 400 °C.

#### 4.1.3. *f* = 5 Hz

**Figure 7:**
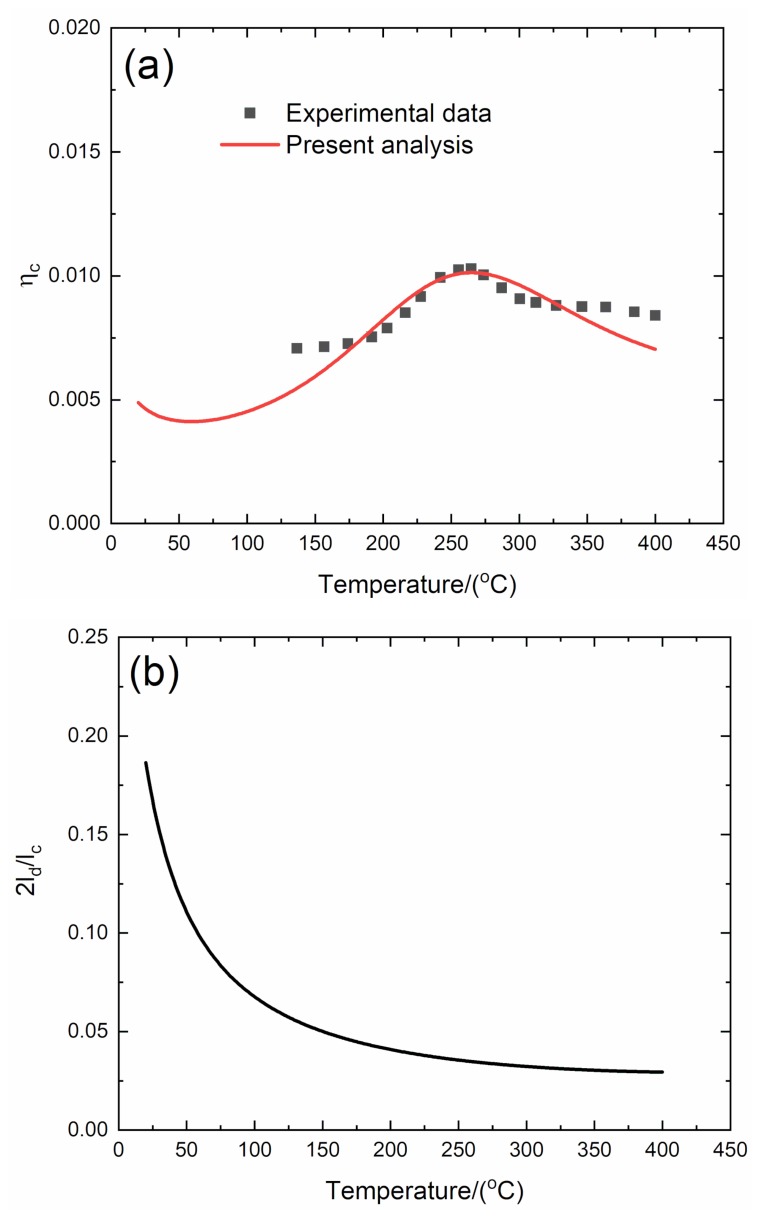

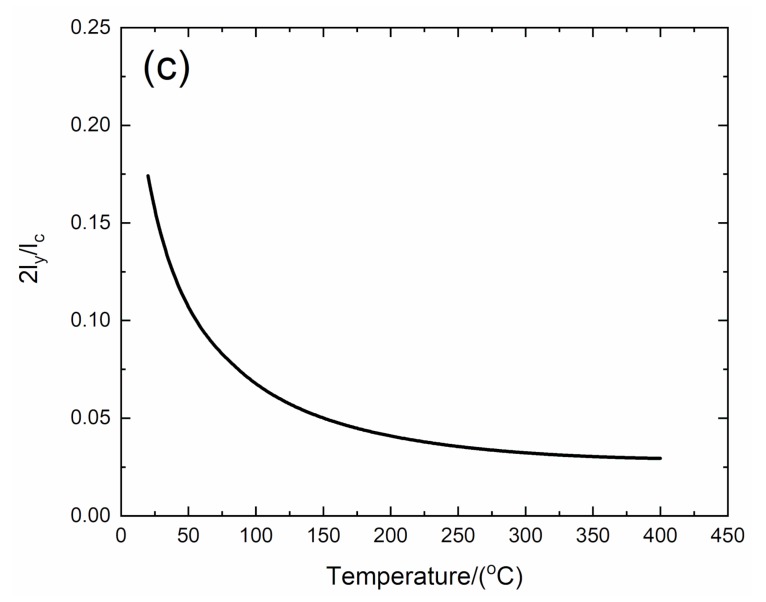
(**a**) Experimental and predicted temperature-dependent composite damping (*η*_c_) versus temperature curves; (**b**) the temperature-dependent interface debonding length (2*l*_d_/*l*_c_) versus temperature curves; and (**c**) the temperature-dependent interface slip length (2*l*_y_/*l*_c_) versus temperature curves of 2D C/SiC composite at the vibration frequency of *f* = 5 Hz.

The experimental and predicted temperature-dependent composite damping (*η*_c_), interface debonding, and slip length (2*l*_d_/*l*_c_, 2*l*_y_/*l*_c_) versus temperature curves of the 2D C/SiC composite at the vibration frequency of *f* = 5 Hz are shown in Figure 7 and Table 5.

The experimental composite damping (*η*_c_) increases from *η*_c_ = 0.007 at temperature of *T* = 136 °C to the peak value of *η*_c_ = 0.0106 at temperature of *T* = 261 °C and then decreases to *η*_c_ = 0.008 at temperature of *T* = 400 °C. The theoretical predicted composite damping increases from *η*_c_ = 0.0048 at room temperature to the peak value of *η*_c_ = 0.0101 at temperature of *T* = 263 °C and then decreases to *η*_c_ = 0.007 at temperature of *T* = 400 °C. The interface debonding length (2*l*_d_/*l*_c_) decreases from 2*l*_d_/*l*_c_ = 0.186 at room temperature to 2*l*_d_/*l*_c_ = 0.029 at temperature of *T* = 400 °C, and the interface slip length (2*l*_y_/*l*_c_) decreases from 2*l*_y_/*l*_c_ = 0.174 at room temperature to 2*l*_y_/*l*_c_ = 0.029 at temperature of *T* = 400 °C.

#### 4.1.4. *f* = 10 Hz

**Figure 8:**
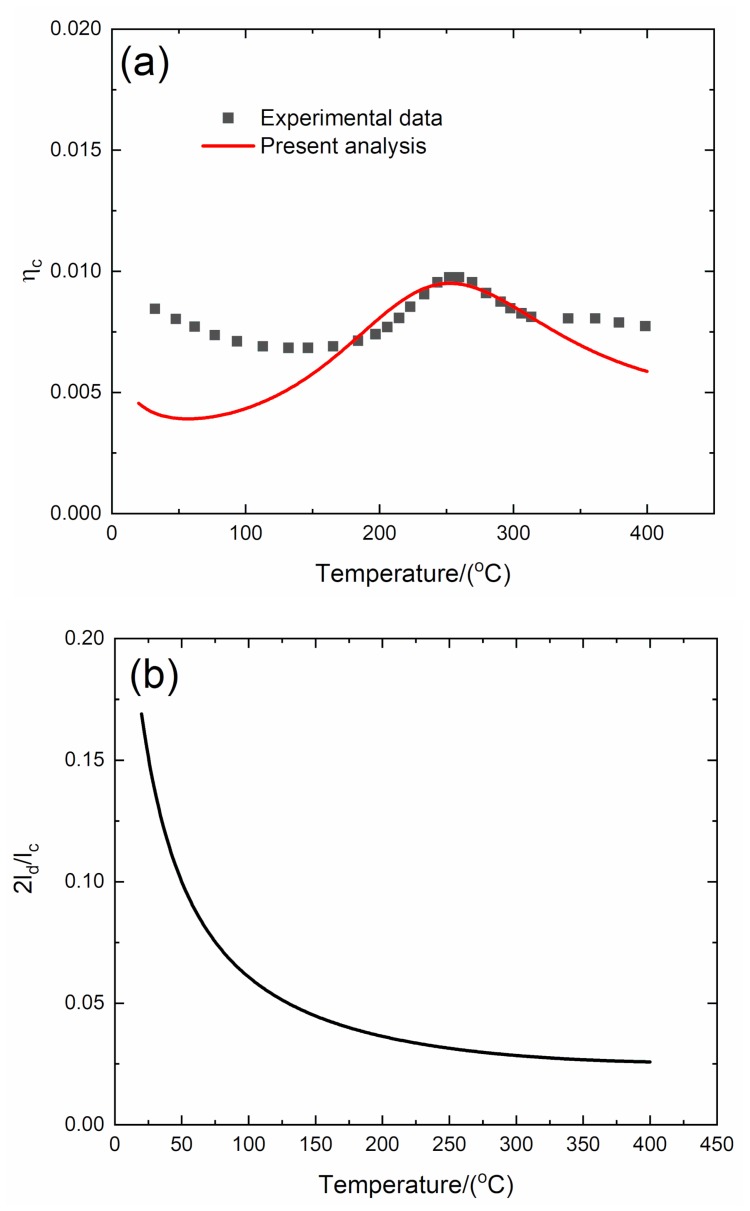

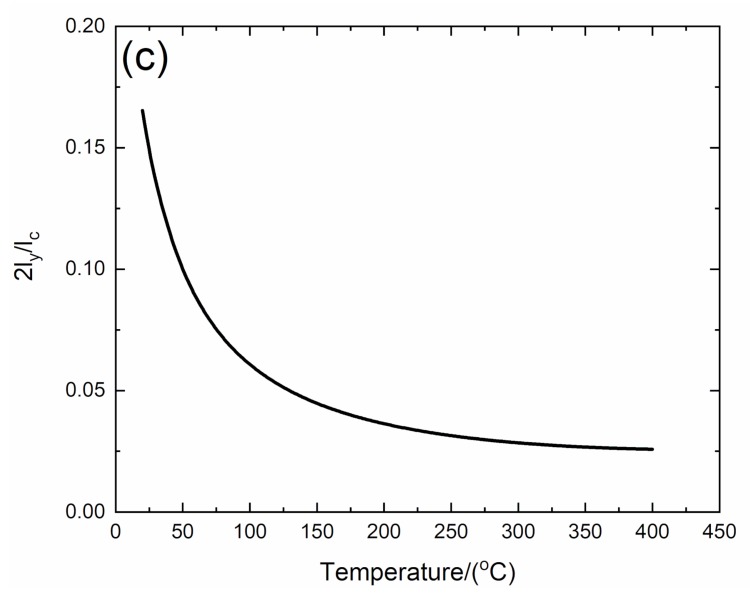
(**a**) Experimental and predicted temperature-dependent composite damping (η_c_) versus temperature curves; (**b**) the temperature-dependent interface debonding length (2l_d_/l_c_) versus temperature curves; and (**c**) the temperature-dependent interface slip length (2l_y_/l_c_) versus temperature curves of 2D C/SiC composite at the vibration frequency of *f* = 10 Hz.

The experimental and predicted temperature-dependent composite damping (*η*_c_), interface debonding, and slip length (2*l*_d_/*l*_c_, 2*l*_y_/*l*_c_) versus temperature curves of the 2D C/SiC composite at the vibration frequency of *f* = 10 Hz are shown in Figure 8 and Table 5.

The experimental composite damping (*η*_c_) decreases from *η*_c_ = 0.0085 at room temperature to *η*_c_ = 0.0068 at temperature of *T* = 125 °C, then increases to the peak value of *η*_c_ = 0.01 at temperature of *T* = 258 °C, and then decreases to *η*_c_ = 0.007 at temperature of *T* = 400 °C. The theoretical predicted composite damping (*η*_c_) increases from *η*_c_ = 0.0045 at room temperature to the peak value of *η*_c_ = 0.0095 at temperature of *T* = 256 °C and then decreases to *η*_c_ = 0.0058 at temperature of *T* = 400 °C. The interface debonding length (2*l*_d_/*l*_c_) decreases from 2*l*_d_/*l*_c_ = 0.169 at room temperature to 2*l*_d_/*l*_c_ = 0.025 at temperature of *T* = 400 °C, and the interface slip length (2*l*_y_/*l*_c_) decreases from 2*l*_y_/*l*_c_ = 0.165 at room temperature to 2*l*_y_/*l*_c_ = 0.0258 at temperature of *T* = 400 °C.

Under a high loading frequency of *f* = 10 Hz, the damage mechanism of CMCs including matrix cracking and interface debonding are affected by the loading frequency. Sorensen and Holmes [29] investigated the effect of loading rate on tensile behavior of a SiC/CAS II composite. It was found that the saturation matrix crack spacing increases with loading rate, and dynamic frictional coefficient also increases. However, in the present analysis, the effect of temperature on dynamic loading damage of CMCs (i.e., matrix cracking and interface damage) is not considered. The predicted composite damping is different from the experimental result at low temperature.

### 4.2. 3D C/SiC Composite

#### 4.2.1. *f* = 1 Hz

**Figure 9:**
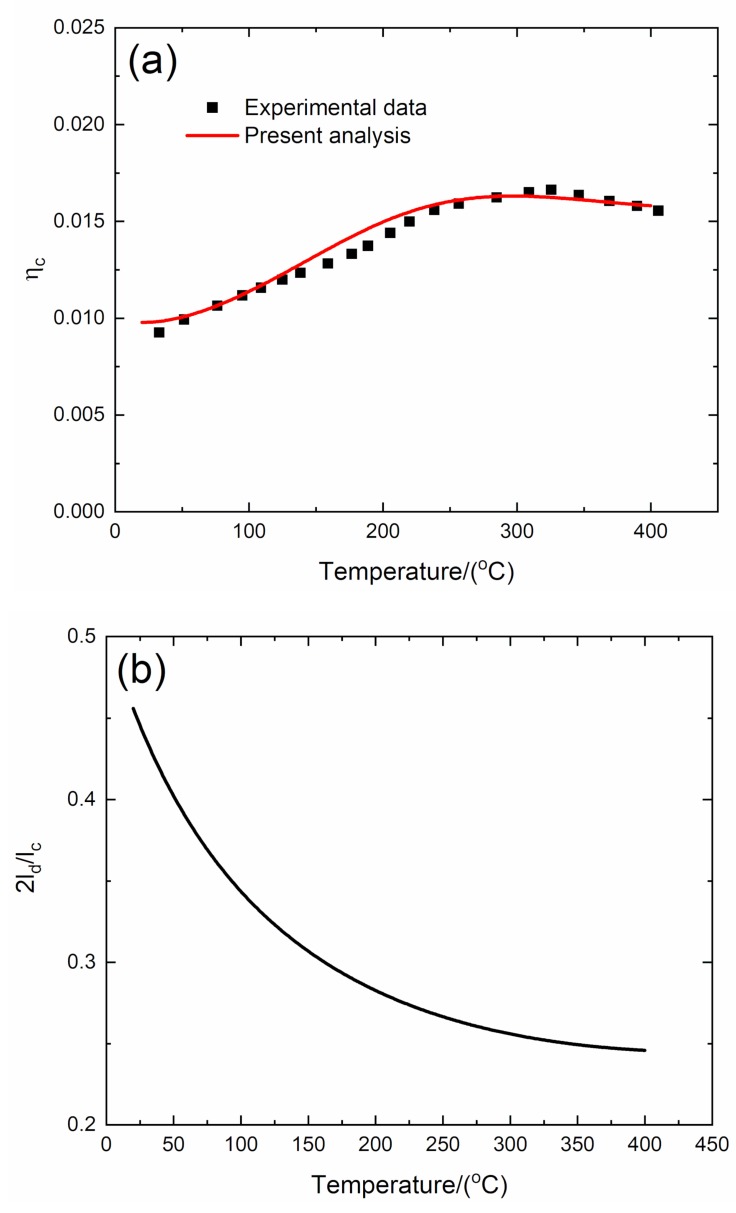

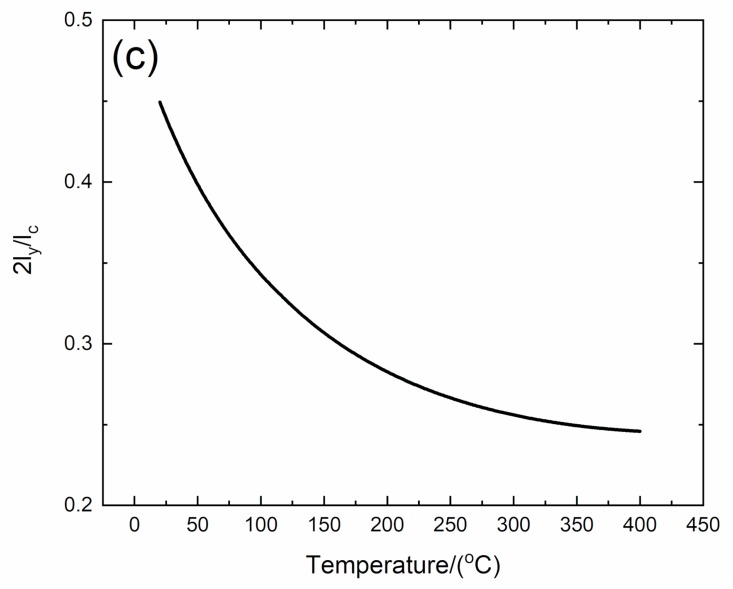
(**a**) Experimental and predicted temperature-dependent composite damping (*η*_c_) versus temperature curves; (**b**) the temperature-dependent interface debonding length (2*l*_d_/*l*_c_) versus temperature curves; and (**c**) the temperature-dependent interface slip length (2*l*_y_/*l*_c_) versus temperature curves of 3D C/SiC composite at the vibration frequency of *f* = 1 Hz.

The experimental and predicted temperature-dependent composite damping (*η*_c_), interface debonding, and slip length (2*l*_d_/*l*_c_, 2*l*_y_/*l*_c_) versus temperature curves of the 3D C/SiC composite at the vibration frequency of *f* = 1 Hz are shown in Figure 9 and Table 6.

The experimental composite damping (*η*_c_) increases from *η*_c_ = 0.009 at room temperature to the peak value of *η*_c_ = 0.0165 at temperature of *T* = 325 °C and then decreases to *η*_c_ = 0.015 at temperature of *T* = 400 °C. The theoretical predicted composite damping (*η*_c_) increases from *η*_c_ = 0.009 at room temperature to the peak value of *η*_c_ = 0.0163 at temperature of *T* = 308 °C and then decreases to *η*_c_ = 0.015 at temperature of *T* = 400 °C. The interface debonding length (2*l*_d_/*l*_c_) decreases from 2*l*_d_/*l*_c_ = 0.455 at room temperature to 2*l*_d_/*l*_c_ = 0.245 at temperature of *T* = 400 °C; and the interface slip length (2*l*_y_/*l*_c_) decreases from 2*l*_y_/*l*_c_ = 0.449 at room temperature to 2*l*_y_/*l*_c_ = 0.245 at temperature of *T* = 400 °C.

#### 4.2.2. *f* = 2 Hz

**Figure 10:**
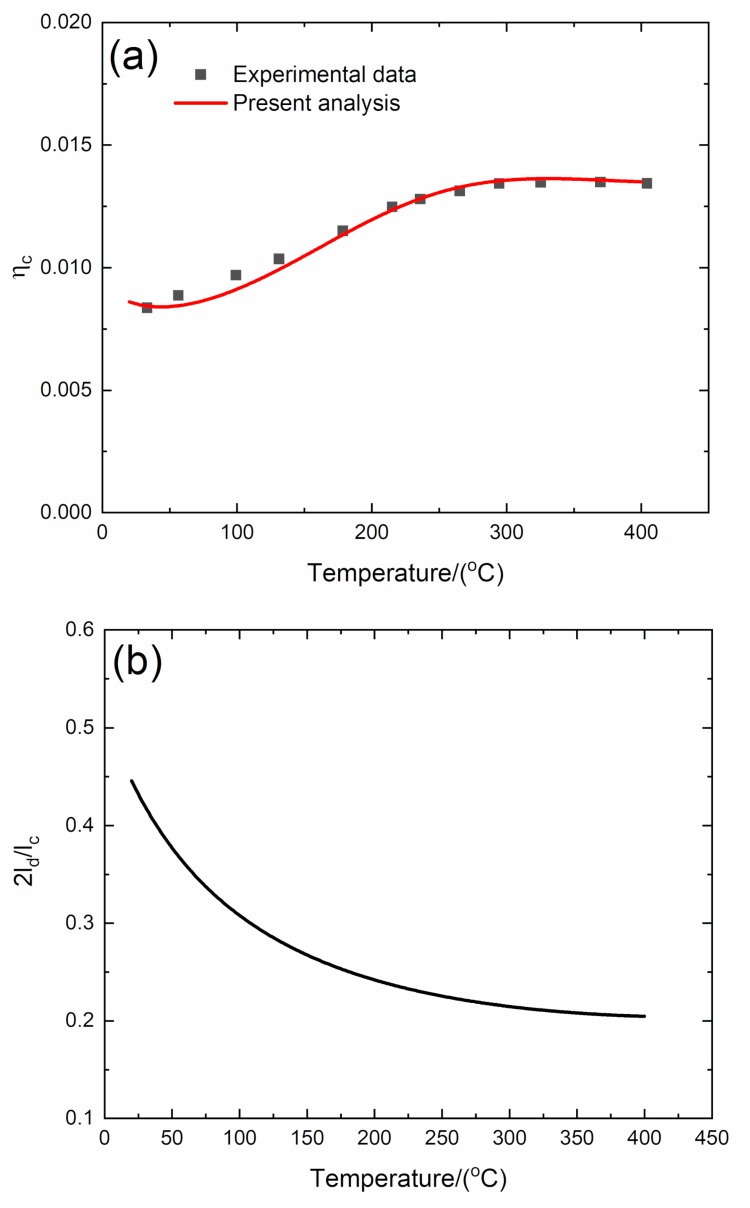

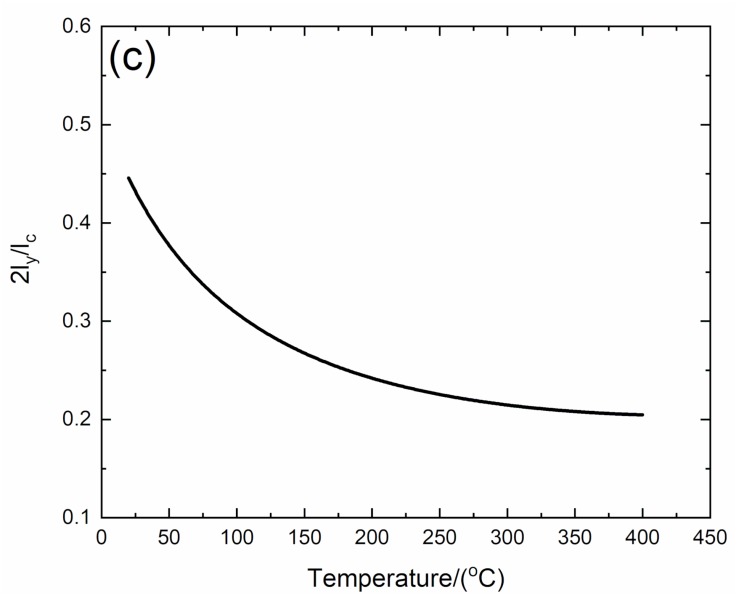
(**a**) Experimental and predicted temperature-dependent composite damping (*η*_c_) versus temperature curves; (**b**) the temperature-dependent interface debonding length (2*l*_d_/*l*_c_) versus temperature curves; and (**c**) the temperature-dependent interface slip length (2*l*_y_/*l*_c_) versus temperature curves of 3D C/SiC composite at the vibration frequency of *f* = 2 Hz.

The experimental and predicted temperature-dependent composite damping (*η*_c_), interface debonding, and slip length (2*l*_d_/*l*_c_, 2*l*_y_/*l*_c_) versus temperature curves of the 3D C/SiC composite at the vibration frequency of *f* = 2 Hz are shown in Figure 10 and Table 6.

The experimental composite damping (*η*_c_) increases from *η*_c_ = 0.0083 at room temperature to the peak value of *η*_c_ = 0.0135 at temperature of *T* = 370 °C and then decreases to *η*_c_ = 0.0134 at temperature of *T* = 400 °C. The theoretical predicted composite damping (*η*_c_) increases from *η*_c_ = 0.0086 at room temperature to the peak value of *η*_c_ = 0.0136 at temperature of *T* = 360 °C and then decreases to *η*_c_ = 0.0135 at temperature of *T* = 400 °C. The interface debonding length (2*l*_d_/*l*_c_) decreases from 2*l*_d_/*l*_c_ = 0.445 at room temperature to 2*l*_d_/*l*_c_ = 0.204 at temperature of *T* = 400 °C; and the interface slip length (2*l*_y_/*l*_c_) decreases from 2*l*_y_/*l*_c_ = 0.445 at room temperature to 2*l*_y_/*l*_c_ = 0.204 at temperature of *T* = 400 °C.

#### 4.2.3. *f* = 5 Hz

**Figure 11:**
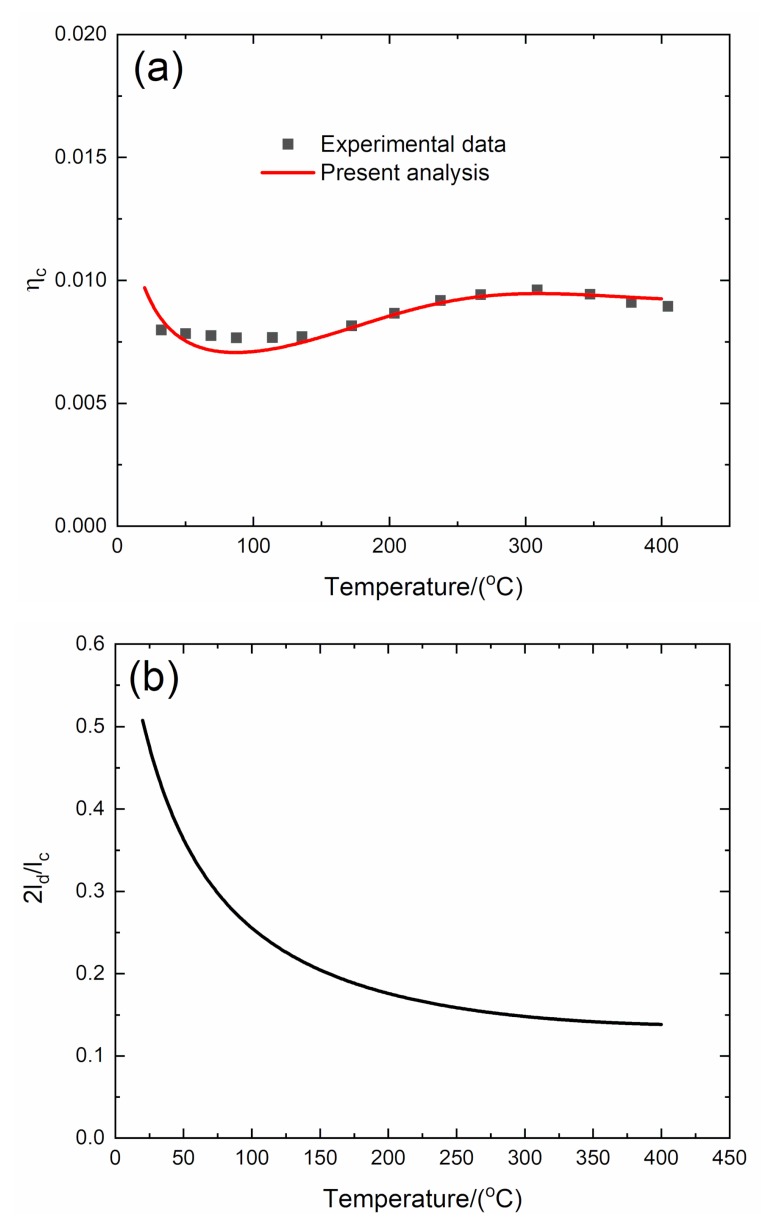

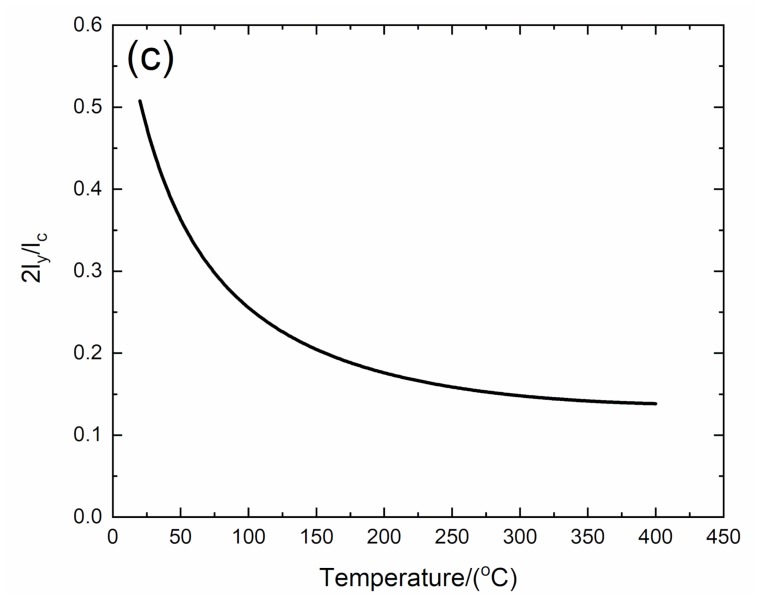
(**a**) Experimental and predicted temperature-dependent composite damping (*η*_c_) versus temperature curves; (**b**) the temperature-dependent interface debonding length (2*l*_d_/*l*_c_) versus temperature curves; and (**c**) the temperature-dependent interface slip length (2*l*_y_/*l*_c_) versus temperature curves of 3D C/SiC composite at the vibration frequency of *f* = 5 Hz.

The experimental and predicted temperature-dependent composite damping (*η*_c_), interface debonding, and slip length (2*l*_d_/*l*_c_, 2*l*_y_/*l*_c_) versus temperature curves of the 3D C/SiC composite at the vibration frequency of *f* = 5 Hz are shown in Figure 11 and Table 6.

The experimental composite damping (*η*_c_) increases from *η*_c_ = 0.008 at room temperature to the peak value of *η*_c_ = 0.0095 at temperature of *T* = 300 °C and then decreases to *η*_c_ = 0.009 at temperature of *T* = 400 °C. The theoretical predicted composite damping (*η*_c_) decreases from *η*_c_ = 0.0097 at room temperature to *η*_c_ = 0.007 at temperature of *T* = 86 °C, then increases to the peak value of *η*_c_ = 0.0095 at temperature of *T* = 300 °C, and then decreases to *η*_c_ = 0.0092 at temperature of *T* = 400 °C. The interface debonding length (2*l*_d_/*l*_c_) decreases from 2*l*_d_/*l*_c_ = 0.507 at room temperature to 2*l*_d_/*l*_c_ = 0.138 at temperature of *T* = 400 °C; and the interface slip length (2*l*_y_/*l*_c_) decreases from 2*l*_y_/*l*_c_ = 0.507 at room temperature to 2*l*_y_/*l*_c_ = 0.138 at temperature of *T* = 400 °C.

#### 4.2.4. *f* = 10 Hz

**Figure 12:**
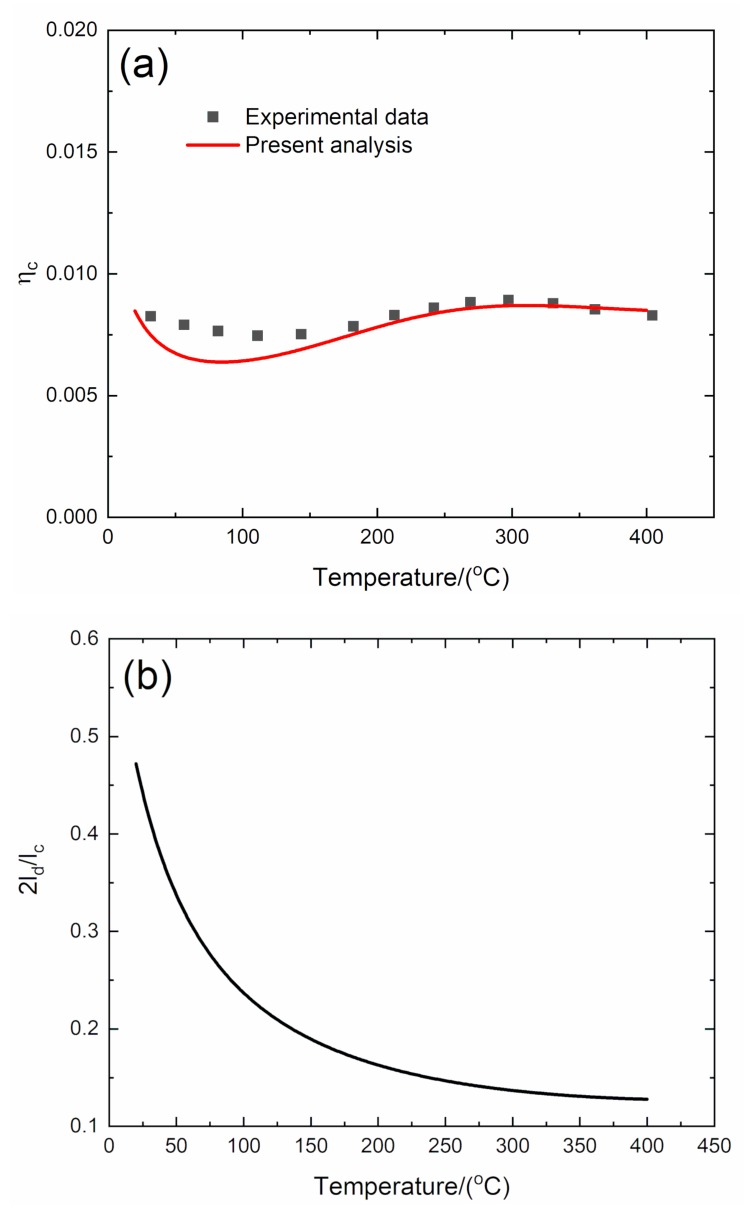

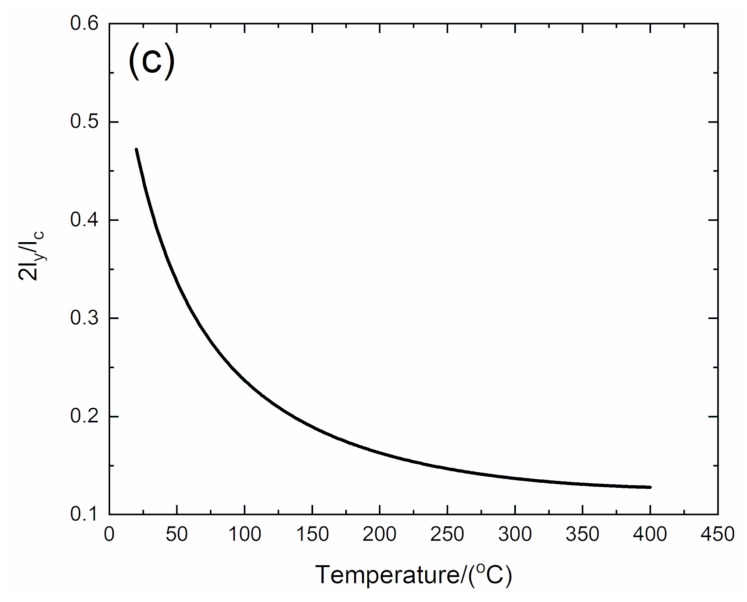
(**a**) Experimental and predicted temperature-dependent composite damping (*η*_c_) versus temperature curves; (**b**) the temperature-dependent interface debonding length (2*l*_d_/*l*_c_) versus temperature curves; and (**c**) the temperature-dependent interface slip length (2*l*_y_/*l*_c_) versus temperature curves of 3D C/SiC composite at the vibration frequency of *f* = 10 Hz.

The experimental and predicted temperature-dependent composite damping (*η*_c_), interface debonding, and slip length (2*l*_d_/*l*_c_, 2*l*_y_/*l*_c_) versus temperature curves of the 3D C/SiC composite at the vibration frequency of *f* = 10 Hz are shown in Figure 12 and Table 6.

The experimental composite damping (*η*_c_) decreases from *η*_c_ = 0.0084 at room temperature to *η*_c_ = 0.0075 at temperature of *T* = 125 °C, then increases to the peak value of *η*_c_ = 0.009 at temperature of *T* = 295 °C, and then decreases to *η*_c_ = 0.0084 at temperature of *T* = 400 °C. The theoretical predicted composite damping (*η*_c_) decreases from *η*_c_ = 0.0084 at room temperature to *η*_c_ = 0.0064 at temperature of *T* = 96 °C, then increases to the peak value of *η*_c_ = 0.0087 at temperature of *T* = 300 °C, and then decreases to *η*_c_ = 0.0085 at temperature of *T* = 400 °C. The interface debonding length (2*l*_d_/*l*_c_) decreases from 2*l*_d_/*l*_c_ = 0.472 at room temperature to 2*l*_d_/*l*_c_ = 0.127 at temperature of *T* = 400 °C; and the interface slip length (2*l_y_*/*l*_c_) decreases from 2*l*_y_/*l*_c_ = 0.472 at room temperature to 2*l*_y_/*l*_c_ = 0.127 at temperature of *T* = 400 °C.

### 4.3. Discussion

Due to temperature-dependent material properties and especially the interface properties (i.e., the interface shear stress (τ_i_(*T*))), the composite damping, interface debonding, and slip state of C/SiC are temperature-dependent. For 2D and 3D C/SiC, the temperature-dependent composite vibration damping increases with temperature to the peak value and then decreases; and the temperature-dependent interface debonding and slip length decrease with temperature. The experimental and predicted composite damping peak values of 2D and 3D C/SiC under vibration frequencies of f = 1, 2, 5, and 10 Hz from room temperature to 400 °C are shown in Table 5 and Table 6.

For 2D C/SiC, the composite damping peak value decreases with vibration frequency, i.e., from *η*_c_ = 0.019 at a vibration frequency of f = 1 Hz to *η*_c_ = 0.01 at a vibration frequency of f = 10 Hz, and the corresponding temperature for peak composite damping also decreases, i.e., from *T* = 283 °C at a vibration frequency of f = 1 Hz to *T* = 258 °C at a vibration frequency of f = 10 Hz.

For 3D C/SiC, the composite damping peak value decreases with vibration frequency, i.e., from *η*_c_ = 0.0165 at a vibration frequency of f = 1 Hz to *η*_c_ = 0.009 at a vibration frequency of f = 10 Hz, and the corresponding temperature for peak composite damping also decreases, i.e., from *T* = 325 °C at a vibration frequency of f = 1 Hz to *T* = 295 °C at a vibration frequency of f = 10 Hz.

For C/SiC, the fiber and matrix damping contributes little to composite damping. However, the frictional dissipated energy caused by frictional slip in the debonding region mainly contributes to the composite damping. For 2D C/SiC, when the vibration frequency increases, the dynamic frictional slip range (i.e., the interface debonding length 2*l*_d_/*l*_c_ and interface slip length 2*l*_y_/*l*_c_) decreases, which decreases the energy dissipated through frictional slip and composite damping. For C/SiC with weak interface bonding, the interface debonding occurs when matrix cracking propagates to the fiber/matrix interphase. The frictional slip between the fiber and the matrix or between fiber and fiber causes the energy dissipation, which contributes to the damping of C/SiC. However, when the interface slip range or interface debonding/slip length decreases, the composite damping decreases.

For C/SiC, the composite damping of 2D C/SiC is higher than that of 3D C/SiC, mainly due to the damage mechanisms of matrix cracking and interface debonding. For 3D C/SiC, the fiber volume along the longitudinal loading direction is higher than that of 2D C/SiC, leading to higher matrix cracking density, low interface debonding length, and low composite damping.

## 5. Conclusions

In this paper, a micromechanical temperature-dependent vibration damping model of a C/SiC composite is developed. The composite damping is divided into damping of the fiber and the matrix and the damping caused by frictional dissipated energy. The relationships between composite damping, composite internal damage, and temperature are established for different material properties and damage states. The experimental temperature-dependent damping of 2D and 3D C/SiC are predicted for different vibration frequencies.

(1) For C/SiC, the temperature-dependent composite vibration damping increases with temperature to the peak value and then decreases, and the temperature-dependent interface debonding and slip length decrease with temperature.
(2) For C/SiC, when the vibration frequency increases, the dynamic frictional slip range decreases, which decreases the energy dissipated through frictional slip and composite damping.
(3) For 3D C/SiC, the fiber volume along the longitudinal loading direction is higher than that of 2D C/SiC, leading to higher matrix cracking density, low interface debonding length, and low composite damping.
(4) When fiber volume and interface debonding energy increase, the peak value of composite damping and the corresponding temperature both decrease.
(5) When matrix crack spacing and steady-state interface shear stress increase, the peak value of composite damping decreases, and the corresponding temperature for peak damping changes a little.

## Figures and Tables

**Table 1:** Temperature-dependent composite damping, interface debonding, and slip length of C/SiC composite for different fiber volumes.

	***T*/(°C)**	***η*_c_**	**2*l*_d_/*l*_c_**	**2*l*_y_/*l*_c_**
*V*_f_ = 30%	20	0.00306	0.152	0.15
	262	0.00752	0.056	0.056
	400	0.00527	0.048	0.048
	*T*/(°C)	*η*_c_	2*l*_d_/*l*_c_	2*l*_y_/*l*_c_
*V*_f_ = 35%	20	0.00223	0.1	0.1
	250	0.00431	0.036	0.036
	400	0.00301	0.03	0.03

**Table 2:** Temperature-dependent composite damping, interface debonding, and slip length of C/SiC composite for different matrix crack spacing.

	***T*/(°C)**	***η*_c_**	**2*l*_d_/*l*_c_**	**2*l*_y_/*l*_c_**
*l*_c_ = 300μm	20	0.00238	0.101	0.1
	263	0.0056	0.037	0.037
	400	0.0039	0.032	0.032
	*T*/(°C)	*η*_c_	2*l*_d_/*l*_c_	2*l*_y_/*l*_c_
*l*_c_ = 400μm	20	0.00207	0.0759	0.0751
	263	0.00458	0.0281	0.0281
	400	0.00207	0.0243	0.0243

**Table 3:** Temperature-dependent composite damping, interface debonding, and slip length of C/SiC composite for different interface debonding energy.

	***T*/(** **°C)**	***η*_c_**	**2*l*_d_/*l*_c_**	**2*l*_y_/*l*_c_**
ξ_d_ = 0.2 J/m^2^	20	0.00245	0.0949	0.0949
	256	0.00478	0.0332	0.0332
	400	0.00337	0.0279	0.0279
	*T*/(°C)	*η*_c_	2*l*_d_/*l*_c_	2*l*_y_/*l*_c_
ξ_d_ = 0.3 J/m^2^	20	0.00172	0.0511	0.0511
	245	0.0022	0.0154	0.0154
	400	0.00174	0.0118	0.0118

**Table 4:** Temperature-dependent composite damping, interface debonding, and slip length of C/SiC composite for different steady-state interface shear stress.

	***T*/(°C)**	***η*_c_**	**2*l*_d_/*l*_c_**	**2*l*_y_/*l*_c_**
τ_0_ =4 0 MPa	20	0.00235	0.094	0.094
	264	0.00627	0.043	0.043
	400	0.00448	0.038	0.038
	*T*/(°C)	*η*_c_	2*l*_d_/*l*_c_	2*l*_y_/*l*_c_
τ_0_ = 50 MPa	20	0.00202	0.0663	0.0663
	265	0.00535	0.0345	0.0345
	400	0.00389	0.0309	0.0309

**Table 5:** Experimental and predicted peak value of composite damping and corresponding temperature of 2D C/SiC composite under the vibration frequencies of f = 1, 2, 5, and 10 Hz at temperature range from room temperature to 400 °C.

Frequency/Hz	Experiment [15]		Theory	
	Peak Damping	Temperature/(°C)	Peak Damping	Temperature/(°C)
1	0.019	283	0.019	279
2	0.015	266	0.014	283
5	0.0106	261	0.0101	263
10	0.010	258	0.0095	256

**Table 6:** Experimental and predicted peak value of composite damping and corresponding temperature of 3D C/SiC composite under the vibration frequencies of f = 1, 2, 5, and 10 Hz at temperature range from room temperature to 400 °C.

Frequency/Hz	Experiment [15]		Theory	
	Peak Damping	Temperature/(°C)	Peak Damping	Temperature/(°C)
1	0.0165	325	0.0163	308
2	0.0135	370	0.0136	360
5	0.0095	300	0.0095	300
10	0.009	295	0.0087	300

## Data Availability

The data used to support the findings of this study are available from the paper.

## Nomenclature

η	composite damping
η_a_	composite damping of CMCs without damage
η_b_	composite damping of CMCs with damage
η_c_	total composite damping of CMCs
U_d_	dissipated energy density per cycle
U	maximum strain energy density per cycle
σ	stress amplitude of vibration stress
σ_c_	vibration stress
ω	vibration frequency
r_f_	fiber radius
V_f_	fiber volume
V_m_	matrix volume
E_f_	fiber elastic modulus
E_m_	matrix elastic modulus
${\bar{E}}_{m}$	effective matrix elastic modulus
E_c_	composite elastic modulus
l_d_	interface debonding length
l_y_	interface counter slip length
l_z_	interface new slip length
l_c_	matrix crack spacing
U_d_u_	dissipated energy density upon unloading
U_d_r_	dissipated energy density upon reloading
U_f_	fiber strain energy density per cycle
U_m_	matrix strain energy density per cycle
ξ_d_	interface debonding energy
ρ	shear-lag model parameter
σ_fo_	fiber axial stress in the bonding region
σ_mo_	matrix axial stress in the bonding region
α_rf_	fiber radial thermal expansional coefficient
α_lf_	fiber axial thermal expansional coefficient
α_rm_	matrix radial thermal expansional coefficient
α_lm_	matrix axial thermal expansional coefficient
α_lc_	composite axial thermal expansional coefficient
ΔT	temperature difference between testing and fabricated temperature
τ_i_	interface shear stress
τ_0_	steady-state interface shear stress
μ	interface frictional coefficient
